# Genomic approaches for studying crop evolution

**DOI:** 10.1186/s13059-018-1528-8

**Published:** 2018-09-21

**Authors:** Mona Schreiber, Nils Stein, Martin Mascher

**Affiliations:** 1 Leibniz Institute of Plant Genetics and Crop Plant Research (IPK) Gatersleben, Corrensstraße 3, 06466 Seeland, Germany; 2 German Centre for Integrative Biodiversity Research (iDiv) Halle-Jena-Leipzig, Deutscher Platz 5e, 04103 Leipzig, Germany

## Abstract

Understanding how crop plants evolved from their wild relatives and spread around the world can inform about the origins of agriculture. Here, we review how the rapid development of genomic resources and tools has made it possible to conduct genetic mapping and population genetic studies to unravel the molecular underpinnings of domestication and crop evolution in diverse crop species. We propose three future avenues for the study of crop evolution: establishment of high-quality reference genomes for crops and their wild relatives; genomic characterization of germplasm collections; and the adoption of novel methodologies such as archaeogenetics, epigenomics, and genome editing.

## Introduction

**Fig. 1:**
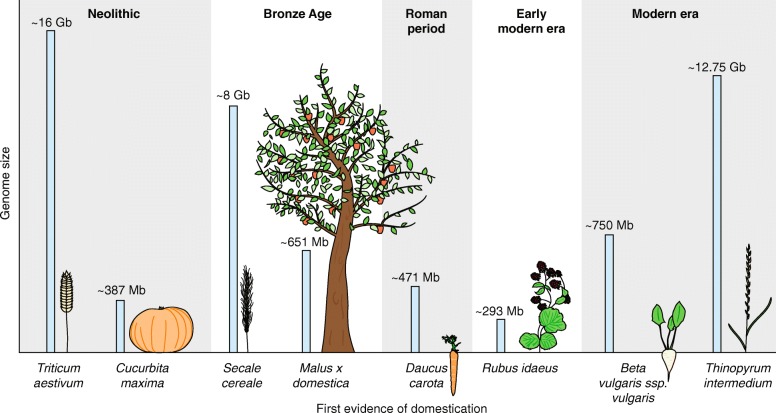
Time of domestication and genome size of domesticated plants.

Since the Neolithic, humans have domesticated a large number of different plant species to create a reliable source of nutrition for themselves and their domestic animals. Crop plants comprise a large variety of species from diverse taxa that differ in habitat, growth habit, and life cycle, such as annual grasses, perennial trees, and medicinal herbs (Table 1, Fig. 1). However, world-wide crop production is dominated by a few major crops, such as wheat, rice, maize, potato, sugar cane, and soybean [1], that serve globally as staples for human and animal nutrition. By contrast, minor crops can be broadly defined as a non-homogeneous group comprising staple crops traditionally only of regional importance, such as quinoa, teff, and African rice; or crops of world-wide importance but comparatively little contribution to human food consumption such as nuts or small fruits. Active research and breeding communities exist for almost every crop plant; however, research into the molecular genetics of domestication has focused mainly on the major crops [2].

**Table 1:** Examples of domesticated crops with domestication origin, available reference genomes and sequencing strategies.

Crop	Botanical name	Lifecycle	Ploidy level	Time of domestication	Geographical origin	Reference genome	Genome size	Sequencing strategy
African rice	*Oryza glaberrima*	Annual	Diploid	~3 000 BP	Upper Niger River?	Wang et al. [200]	~860 Mb	Sanger, Roche/454
Amaranth	*Amaranthus* spp.	Annual	Tetraploid	Aztecs? ~13^th^ – 15^th^ century	Mesoamerica	Clouse et al. [46]	~456 Mb	Illumina, physical map
Apple	*Malus* x *domestica*	Perennial	Diploid; polyploid karyotypes exist	Late Bronze Age? ~2 000 – 1 500 BC	West-Asia	Daccord et al. [157]	~651 Mb	Illumina, PacBio, optical map
Asian rice	*Oryza sativa*	Annual	Diploid	>6 200 BC	China	Kawahara et al. [201]	~500 Mb	Sanger, Illumina, Roche/ 454, optical mapping
Barley	*Hordeum vulgare*	Annual	Diploid	~10 000 BC	Southwest Asia	Mascher et al. [52]	~5 Gb	Illumina, optical mapping, genetic map, HiC
Beet	*Beta vulgaris*	Biennial	Diploid	Before 800 BC	Middle East	Dohm et al. [202]	~750 Mb	Roche/454, Illumina, Sanger
Bread wheat	*Triticum aestivum*	Annual	Hexaploid	~10 000 BC	Southwest Asia	Zimin et al. [65]	~17 Gb	Illumina, PacBio
Carrot	*Daucus carota*	Annual/bie-nnial	Diploid	Classical antiquity	Central Asia (south-west Asia)	Iorizzo et al. [49]	~473 Mb	Illumina, Roche/454, linkage map
Cassava	*Manihot esculenta*	Annual	Diploid	10 000 – 5 000 BC in South America	Amazon basin	Wang et al. [203]	~770 Mb	Illumina, Roche/454
Chickpea	*Cicer arientinum*	Annual	Diploid	~10 000 BC	Fertile Crescent	Varshney et al. [204]	~740 Mb	Illumina
Cotton	*Gossypium hirsutum*	Perennial	Tetraploid	~6 000 – 5 000BC	India/Mexico	Li et al. [205]	~2.4 Gb	Illumina, genetic map
Cowpea	*Vigna unguiculata*	Annual	Diploid	~4 000 BC	West Africa	Muñoz-Amatriaín et al. [206]	~620 Mb	Illumina, physical map, genetic map
Emmer wheat	*Triticum dicoccon*	Annual	Tetraploid	~10 000 BC	Southwest Asia	Avni et al. [58]	~12 Gb	Illumina, genetic map, HiC
Ginseng	*Panax ginseng*	Perennial	Tetraploid	Cultivated since ~3000 BC	China	Jayakodi et al. [207]	~3.5 Gb	Illumina
Intermediate wheatgrass	*Thinopyrum intermedium*	Perennial	Hexaploid	~6 000 BC	Central and south-eastern Europe to Anatolia	No reference available	~12.75 Gb	Genetic map [208]
Maize	*Zea mays*	Annual	Diploid	~6 700 BC	Mexico	Jiao et la. [63]	~2.4 Gb	PacBio, optical mapping, Illumina
Oat	*Avena sativa*	Annual	Hexaploid	European Bronze Age ~1 800 BC	Fertile Crescent	In progress: The Oat Genome Project [209]	~12 Gb	
Pepper	*Capsicum* spp.	Annual	Diploid	~4 000 BC	South and Middle America	Qin et al. [99]	~3.5 Gb	Illumina, 10X, genetic map
Potato	*Solanum tuberosum*	Annual	Autotetraploid	~ 7 000 – 6 000 BC	Andean region	The Potato Genome Sequencing Consortium 2011 [210]	~844 Mb	Illumina, Roche/454, Sanger
Pumpkin	*Cucurbita maxima*	Annual	Tetraploid	~8 000 BC	South America	Sun et al. [47]	~378 Mb	Illumina, genet map
Quinoa	*Chenopodium quinoa*	Annual	Tetraploid	> 5 000 BC	Mesoamerica	Jarvis et al. [32]	~ 1.5 Gb	PacBio, Illumina, optical mapping
Rapeseed	*Brassica napus*	Annual	Tetraploid	~400 – 500 BP	Europe	Yang et al. [211]	~1.13 Gb	Illumina, PacBio, optical mapping
Raspberry	*Rubus idaeus*	Perennial	Tetraploid	~450 BP	Europe and northern Asia	Van Buren et al. [48]	~293 Mb (black raspberry)	Illumina, genetic map
Rye	*Secale cereale*	Annual	Diploid	European Bronze Age ~1 500 – 1 000 BC	Europe	Bauer et al. [212]	~7.9 Gb	Illumina, genetic map
Sorghum	*Sorghum bicolor*	Annual	Diploid	~7 100 – 6 900 BC	Sahel	McCormick et al. [213]	~730 Mb	Illumina, Roche/454, Sanger
Sugar cane	*Saccharum* spp.	Perennial	Allopolyploid	~8 000 BC	Southeast Asia	Riaño-Pachón et al. [214]	~10 Gb	Illumina
Sunflower	*Helianthus annuus*	Annual	Diploid	~6 000 BC	Eastern North America	Badouin et al. [215]	~3.6 Gb	PacBio, genetic map
Tomato	*Solanum lycopersicum*	Annual	Diploid	Before 15^th^ century	Andean region	The Tomato Genome Sequencing Consortium 2012 [216]	~900 Mb	Roche/454, Illumina

Crop domestication has been studied for more than a century and benefited recently from technological innovations in genomics. Comparative analysis of population genomic data of large samples of current and past varieties of crops together with their wild progenitors provides insight into the domestication history of species [3, 4], for example, (i) when and where domestication occurred, (ii) how the domesticates spread to new habitats and which genetic changes accompanied this transition, and (iii) whether gene flow has occurred between the crops and wild relatives. A synthesis of archaeological and population genetic data evidence indicated that the initial stages of domestication in Southwest Asia should be considered a protracted process [5, 6] rather than a rapid evolution of cultivated plants as presumed previously [7–9]. The “democratization” of genomics [10, 11] has now opened new avenues for understanding the genetic consequences of domestication in a much wider range of species from different centers of origin such as Mesoamerica and Africa.

Much has been written on plant domestication. Recent review articles have focused on convergent phenotypic evolution [12], causative mutations affecting phenotypic variation [13, 14], the effect of gene functions on crop adaptation and selection mechanisms [15], the reduction of genetic diversity and the influence of epigenetic modifications [16], the impact of genomic methods in future crop improvement [17], the value of crop wild relatives [18], sequencing ancient plant DNA [19, 20], and general concepts in plant domestication research [21–23]. Here, we focus on the assembly of reference genome sequences for domesticated plants and their wild relatives; surveying sequence diversity in large diversity panels; and the application of novel approaches such as epigenomics, archaeogenetics, and genome editing to plant domestication research.

## High-quality reference sequence assemblies for crops and their wild relatives

Extant crop wild relatives are excellent tools to understand crop evolution and as sources of novel allelic diversity for future crop improvement [24, 25]. The wild gene pool of a crop includes its wild progenitor species, with which it is often fully interfertile, and species from the same or closely related genera that can differ greatly in divergence times and interfertility with the crop [26]. An unbiased assessment of genetic diversity in distantly related taxa using the single reference genome sequence of the domesticate is complicated by sequence divergence, which prevents the alignment of short reads, especially in non-coding regions. Comparison of short reads to a single reference will also not reveal structural variants such as chromosomal inversions and translocations. Thus, reference genome sequence assemblies of crop wild relatives are important tools to understand domestication history.

Genome sequencing and assembly have been applied to many different species for decades. In the context of plant domestication research, genomic resources such as high-quality reference genome sequences of crops and their wild relatives, but also dense genetic and physical maps, have provided the infrastructure for the genetic mapping of loci underlying key domestication traits [27, 28] and their subsequent isolation by map-based cloning [29–32]. Genome sequence assemblies serve as common references for the alignment of resequencing data from diversity panels comprising crops and their wild progenitors [33, 34], thus underpinning genome scans for phenotypic associations and for targets of selection under domestication [3, 35]. In the past, the large size, repeat-rich structure and polyploid nature of many crop genomes have been major impediments to the construction of contiguous sequence assemblies [36]. Here, we summarize recent developments in sequencing technology and computational methods that have contributed to overcoming these long-standing obstacles; provide recent examples for the construction of high-quality reference for crops and their wild relatives; and outline future directions.

**Fig. 2:**
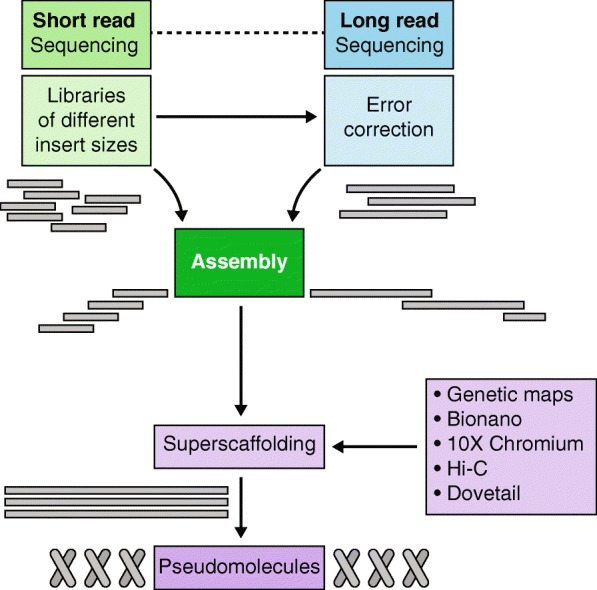
Genome sequence assembly from short-read and long-read data together with genome mapping technologies. Either short or long reads can be used to assemble sequence contigs and scaffolds, which can be ordered along the chromosomes by a battery of super-scaffolding methods.

The traditional approach of laborious and time-consuming Sanger sequencing along a minimum tiling path of bacterial artificial chromosomes (BACs) [37] has been attempted for only a few crops with small genomes such as rice [38] or crops of the highest economic importance—and commensurate research funding—such as maize [39], wheat [40], and barley [41]. In the latter two species, progress in sequencing and genome mapping technologies has prompted the respective international sequencing consortia that set out years ago with physical map construction to revise their strategy by adopting short-read sequencing [42, 43]. Wet-lab protocols and computational methods for genome sequence assembly using short Illumina reads were established about a decade ago [44, 45]. The general approach of genome sequence assembly is shown in Fig. 2 and can be summarized as follows: (i) contig assembly from deep-coverage paired-end reads; (ii) scaffolding with mate-pair information; (iii) filling of sequence gaps introduced in this step; and (iv) high-level ordering of sequence scaffolds into so-called pseudomolecules as representatives of entire chromosomes (‘superscaffolding’). A large number of crop plant genomes have been sequenced using this method, including minor crops such as amaranth [46], pumpkin [47], raspberry [48], and carrot [49]. Genome assemblies for 12 species in the genus *Oryza*, i.e., wild and domesticated rice, and an outgroup species (*Leersia perrieri*) provided a comprehensive overview of structural genome evolution, thus contributing to the overarching aim of the International Oryza Map Alignment Project to establish genus-wide comparative genomics to discover genes for crop improvement [50].

In the large-genome cereals wheat, barley, and rye, short-read sequencing has been widely adopted to compile complementary datasets for higher-order scaffolding such as linkage maps [51, 52], physical maps [53], and chromosome-specific sequences [54]. However, strong reservations persisted against performing initial contig assembly with short reads only. This skepticism was vindicated by the mixed success of initial efforts in wheat and barley. The gene space was reasonably complete and approximately ordered along the genome with the help of genetic and physical maps; however, sequence contiguity was on the kilobase-scale and the repetitive portion of the genome was severely underrepresented [55–57]. Until recently, researchers were unwilling to take the risk of amassing the large amount of sequences required for short-read assembly of multi-gigabase crop genomes without a proven strategy for assembling them.

In an eye-opening study, Avni et al. [58] constructed a chromosome-scale assembly of the genome of tetraploid wild emmer (genome size 10 Gb) from very deep Illumina sequencing data from multiple paired-end, mate-pair libraries and chromosome-conformation capture sequencing data, thus establishing a precedent for the construction of a high-quality sequence assembly of a repeat-rich polyploid plant genome [59]. Several factors contributed to the feasibility and success of their approach, such as improved library construction methods ensuring uniform genome representation [60] and increases in throughput and read length of the Illumina platform (2 × 250 bp) accompanied by decreases in sequencing costs. One important caveat of the work by Avni et al. [58] is that the software used to construct sequence scaffolds with megabase-scale contiguity is the trade secret of a commercial service provider, NRGene. Open-source alternatives for Illumina-only sequence assembly exist and their results exceed assembly metrics of previous efforts by an order of magnitude [61], but they have not yet achieved the contiguity of the wild emmer assembly.

Long reads (> 10 kb) from the PacBio or Oxford Nanopore platforms are two orders of magnitude longer than Illumina reads (100–300 bp) but have reduced sequence accuracy [62]. Recently, PacBio sequencing was used to construct a new version of the maize reference genome [63], which achieved a higher contiguity and better genome representation than the previous BAC-by-BAC assembly [39] and corrected many errors in the order and orientation of sequence contigs of its predecessor. Schmidt et al. [64] used Oxford Nanopore data to assemble the genome sequence of the tomato wild relative *Solanum pennellii* (genome size ~ 1 Gb). The resultant sequence scaffolds were highly contiguous (N50 2.5 Mb) but required correction with supplementary Illumina reads to increase accuracy at the single nucleotide level. Similarly, Zimin et al. [65] used a combination of PacBio and Illumina data to reconstruct the genome sequences of hexaploid bread wheat and its diploid progenitor *Aegilops tauschii* [66]. The N50 values of these assemblies are in the range of several hundred kilobases, which confirms that long-read sequences can produce better assembly than short-read technology even in the most complex genomes, but also highlights the necessity of obtaining short-read sequences for error correction and complementary mid- and long-range information to achieve chromosome-scale contiguity. Another important drawback of using inaccurate long reads are the immense computational requirements of the assembly process (> 100,000 CPU hours for bread wheat [65]).

Until recently, there was a ‘contiguity gap’ in the assembly of complex plant genomes. The contiguity of sequence assemblies was limited to kilobase-sized contigs, but conventional methods for higher-order scaffolding and assembly validation such as genetic mapping [67, 68], cytogenetic methods [69, 70], BAC-based physical mapping [53, 71], or radiation hybrid mapping [72] are effective only at the scale of megabases. Moreover, these techniques are time-consuming and require resources that are difficult or sometimes impossible to generate (e.g., genetic maps in asexual organisms). Methodology has improved dramatically over the past 3 years. Deep-coverage short-read sequencing of paired-end and mate-pair can now deliver contigs of megabase-scale contiguity in complex plant genomes. Furthermore, a plethora of methods—many of them employing Illumina sequencing—has been developed to validate, correct, and improve initial sequence assembly from either long or short reads (Fig. 2). Among the first of these was optical mapping, a method that measures the distances of fluorescently labeled nicking sites in linearized long DNA molecules to construct megabase-scale contigs composed of molecules with matching labeling patterns [73]. Optical mapping on the Bionano platform has been used for scaffolding the PacBio assembly of the maize genome [63] and for higher-order scaffolding of BAC-based or chromosome-specific assemblies in wheat, barley, and tetraploid finger millet [42, 74, 75].

One of the limitations of the current Bionano platform is the need for input assemblies of high contiguity so that sequence scaffolds comprise a sufficient number of label sites to confidently align sequence assembly and optical map. Technologies based on Illumina short-read sequencing can better control data density by increasing sequencing depth. One of these, the Chromium 10X platform, employs a microfluidic device to create so-called ‘linked reads’, which incorporate short barcode sequences that are shared by reads originating from the same high-molecular weight DNA fragment [76]. Chromium reads serve as mid-range linkage information in a similar manner as mate-pair reads or BAC end sequences, but with the added value of longer span size (> 50 kb) and multiple linked short reads that support connections between sequence scaffolds to effectively bridge repetitive regions [77]. Intriguingly, deeply sequenced 10X libraries can be used on their own for genome assembly. After stripping of the barcode from the reads, 10X chromium data can play the role of short-read sequences for assembling by contigs, which are subsequently scaffolded by leveraging information on the molecular origin of reads [78]. Hulse-Kemp et al. [79] used this approach to assemble the genome sequence of bell pepper (*Capsicum annum*) and achieved a scaffold N50 of 3.7 Mb.

Arguably, the most disruptive technology in recent assembly for genome mapping has been chromosome conformation capture sequencing (Hi-C). Hi-C was originally developed as a method to assay genome-wide chromatin contact probabilities [80], but it was soon realized that the linkage information afforded by chromatin proximity can be effectively used for scaffolding fragmented genome assemblies to chromosome-scale contiguity [81, 82]. In addition to ordering and orienting scaffolds, Hi-C can also effectively detect misassemblies. Chromatin contact probabilities between pairs of loci are strongly correlated to their distance in the linear genome [42, 80, 83]. Thus, the likely cause for any strong deviations from the expected rate of distance-dependent decay of contact probabilities are misassemblies [84]. Hi-C mapping made it possible to order sequence scaffolds in the large (> 300 Mb) non-recombining proximal regions of the barley [42] and tetraploid wheat [58] genomes, for which the construction of high-resolution molecular marker maps of high density had remained elusive [85]. Lightfoot et al. [86] used a combination of Hi-C and PacBio long reads to increase the contiguity of the genome assembly of amaranth to chromosome-scale scaffolds. The principle of proximity ligation also underlies the Chicago method, which derives linkage information from Hi-C libraries constructed from chromatin reconstituted in vitro from high molecular weight DNA [87] and is offered commercially by Dovetail Genomics. Dovetail scaffolding was used to improve the assembles of model plants [88, 89], such as lettuce [90], quinoa [32], and an individual chromosome of hexaploid wheat [91].

We believe it is possible to obtain for any plant taxon—wild or domesticated—a high-quality reference genome sequence assembly within a year’s time frame without prior resources. But, which is the most cost-effective combination of sequencing methods and genome mapping approaches [88, 89]? A contiguous, complete, and correctly annotated reference sequence will support research into the contribution of ancestral diversity in the wild progenitors, the footprints of artificial selection in the domesticate, and gene flow between wild and cultivated taxa.

## Genomic characterization of germplasm collections

The wild progenitors of most crop plants remain extant [92] and can be collected from the wild. Furthermore, traditional landraces have been collected and stored in germplasm collections (so called ‘genebanks’ or ‘seed banks’) for more than a century. Germplasm collections can provide the raw material for population genomic studies to unravel the origin of crops, their demographic history, as well as past and present selection pressures. Several strategies based on high-throughput sequencing are available to catalogue and analyze genetic variation in crop diversity panels, namely whole-genome sequencing, exome capture, RNA sequencing, and reduced representation resequencing. The key difference between these approaches lies in the fraction of the genomes targeted for sequencing, which determines the requirements for prior resources and the per sample cost (Table 2).

**Table 2:** Comparison of different resequencing strategies.

	Reference needed?	Complexity reduction	Access to non-coding regions	Cost per sample	Advantage	Disadvantages	Examples
Whole genome resequencing	+++		++++	++++	- Complete representation of the genome - Analysis of structure possible	Expensive	Date palm [217], Maize [218], Pepper [99], Rice [208], Sorghum [219], Soybean [220], Tomato [221]
Exome capture	+++	++		+++	- Sequence of protein coding regions & functional elements - Known target regions	- Only exons - Prior probe design required	Barley [112], Bread wheat [222], Cotton [223], Pine [224], Rapeseed [225], Soybean [226], Sugarcane [227]
RNAseq	++	++++		++	- Gene expression analysis possible - Only protein coding regions - Reference-free analysis methods exist	- Only exons - Only protein coding regions - Coverage depends on transcript abundance	African rice[117], Carrot [228] Common bean [229], Cotton [230], Pepper[99], Sunflower[140], Tomato[34]
Genotyping-by-sequencing	++	++++	+++	+	- High-throughput identification of whole-genome markers - Reference-free analysis methods exist	- Sparse marker data - Handling of missing data required	Cassava[138], Chickpea [231], Cotton[69], Cowpea [232], Oat [233], Soybean [234], Watermelon [235]

The most straightforward method for assessing genetic diversity in species with a reference genome is shotgun sequencing of short-insert libraries on the Illumina platform and alignment of the sequence reads to the reference assembly [93]. Whole-genome shotgun sequencing has been used in major crops such as maize [3, 94], rice [33, 95, 96], soybean [97], and Solanaceous species [98, 99] to study genome-wide sequence diversity in the crop and its wild relatives with a focus on domestication history and the genetic basis of crop improvement. In rice, whole-genome sequencing and phenotyping of diversity panels, together with efficient transgenic methods for testing candidate gene function, has emerged as a powerful approach for isolating genes of agronomic importance [95, 100]. Deep-coverage whole-genome sequencing data of multiple individuals is a prerequisite for advanced population genetic methods to infer demographic history such as the Pairwise-Sequentially Markovian Coalescent model [101] and its derivatives [102, 103], which were used to study historic changes of population size in maize [104], grapevine [105], African rice [106], and their respective wild progenitors.

An important drawback of whole-genome sequencing is the financial burden associated with amassing sufficient sequence data for large diversity panels in minor crops or those with large genomes. Several techniques have been developed to reduce genome complexity prior to sequencing so as to increase read depth in certain genomic regions. Sequence capture with oligonucleotide baits can be applied at the scale of whole exome [107] or gene families [108]. For example, whole-exome capture assays have been developed in wheat [109, 110] and barley [111] and applied in population genetic studies. Russell et al. [112] analyzed exome sequences from > 250 wild and domesticated barleys to understand contrasting patterns of diversity in both taxa and to study changes in haplotype structure of flowering time genes during range expansion. Similarly, Avni et al. [58] used exome capture data from 65 accessions of wild and domesticated emmer to detect genomic regions under selection. Resistance gene enrichment sequencing (RenSeq) was originally developed to enable rapid mapping of nucleotide binding-site leucine-rich repeat resistance genes in mutant collections and segregation populations [108, 113], but has been recently adopted by Arora et al. [114] for association genetics and applied to gene isolation in *Aegilops tauschii*, a wild diploid progenitor of bread wheat. Witek et al. [115] combined resistance gene enrichment sequencing with PacBio sequencing to clone a resistance gene against potato late blight disease in *Solanum americanum*, a diploid, non-tuber bearing wild relative of potato.

High-throughput RNA sequencing (RNA-seq) [116] is an alternative to whole-exome capture that does not require the design of oligonucleotide baits and can assess sequence variation for a large portion of the transcribed part of the genome. For example, Nabholz et al. [117] used RNA-seq to study domestication bottlenecks in African rice. As a method to quantify transcript abundance, RNA sequencing affords information on gene expression levels in addition to genetic variation. Koenig et al. [34] performed RNA-seq on tomato and related *Solanum* species to detect footprints of selection based on genetic variation at the DNA sequence level, and also found selection pressure on gene expression level in the domesticate. Lemmon et al. [118] analyzed allele-specific expression in F_1_ crosses between maize and teosinte to understand the changes in the regulatory architecture of gene expression as a consequence of domestication.

Reduced representation sequencing [119] is known by many names such as RAD-seq [120], genotying-by-sequencing (GBS) [121], or SLAF-seq [122]. The common denominator of all these methods is genomic complexity reduction by digestion with restriction enzymes and subsequent short-read sequencing of fragments bordering restriction sites. We will now use the term ‘genotyping-by-sequencing (GBS)’ as it captures the essence of these methods: they do not produce resequencing data for a gene set, but apply high-throughput sequencing to obtain dense genome-wide marker data. As such, GBS is similar to genotyping with SNP chips. In contrast to SNP chips, however, no prior knowledge and expenses are required to develop genotyping assays and ascertainment bias [123] is reduced. Challenges in the analysis of GBS data include allelic dropout [124] and handling of missing data [125, 126]. In principle, GBS data can be analyzed without a reference genome [127, 128], but care needs to be taken in the interpretation of estimates of population genetic parameters [129].

GBS is a versatile and inexpensive method for natural and experimental populations of crops and their wild relatives. Linkage maps for wild relatives of Triticeae crops [130–132] and crop–wild cross in tomato [133] have been constructed in recent years. Moreover, GBS has been used to characterize crop–wild introgression lines in barley [134] and tomato [135]. In addition to the analysis of experimental populations, GBS has been widely adopted for population genetic studies. For example, it has been used to compare diversity between crops and their wild progenitors in chickpea [136], amaranth [137], and cassava [138]; to study geneflow between wild and domesticated sunflower taxa [139, 140]; and to elucidate the demographic history of wild *Phaseolus vulgaris*, the progenitor of common bean [141].

Moreover, GBS is a cost-effective method to screen large germplasm collections. For example, Romay et al. [142] used GBS to study genetic variation in 2815 maize inbred lines maintained at the US national seedbank. If used at the scale of entire collections, GBS holds the potential of developing molecular passport data for gene bank management, complementing traditional morphological markers and field evaluation. This can help resolve issues regarding (i) duplication within and between gene banks around the world [143], (ii) the genetic integrity of accessions after decades of ex situ maintenance [144, 145], and (ii) the development of truly representative core collections [146] to be subjected to whole-genome sequencing [147] and in-depth phenotyping. The power of this approach was exemplified by a recent study in maize. Navarro et al. [148] genotyped and phenotyped a panel of > 4000 F_1_ hybrids between elite breeding material and diverse landraces sampled from the collection of the International Maize and Wheat Improvement Center (CIMMYT). Genome-wide association scans revealed a co-association of genes to both flowering time and altitude. Similar approaches are underway in other cereal crops such as wheat [149], barley [150], common bean [151], Solanaceous crops [152], and rice [153]. As more genotypic and phenotypic data for large germ plasm collections accrue, strategies for the effective utilization of plant genetic resources into breeding without inflicting yield losses are needed [154].

## Novel approaches to domestication research

**Fig. 3:**
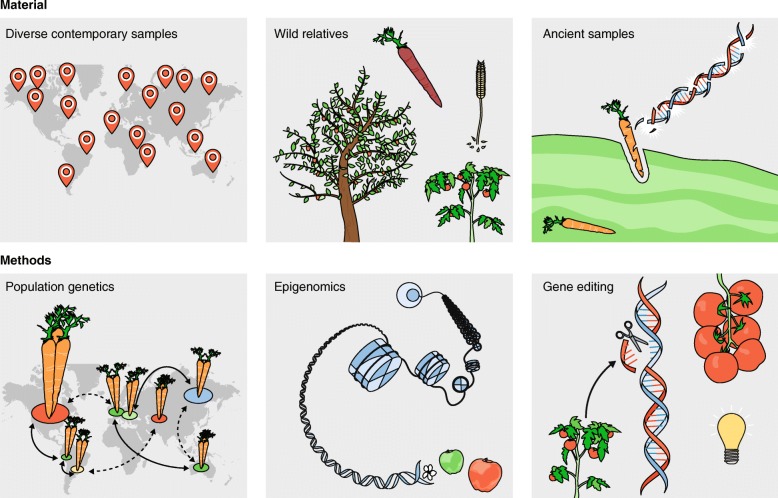
Future directions of domestication research. The study of crop evolution will continue to rely on the population genetic analysis of diversity panels comprising domesticates and their wild relatives. If well-preserved archaeobotanical remains are available, ancient samples can serve as a time-capsule informing about past demography of a crop. Moreover, new approaches such as epigenomics and gene editing will enrich the toolbox of domestication research.

High-throughput sequencing has also been used to obtain measurements on aspects of the genome other than nucleotide sequence variation. Here, we describe future directions in domestication research that will benefit from these technological innovations, such as epigenomics, archaeogenetics, genome editing, domestication of novel crops, and new computational methods for analysis of population genetic data (Fig. 3).

Epigenomics refers to the study of the entirety of heritable changes other than changes in DNA sequence, such as DNA methylation or histone modifications. The interaction between euchromatin and heterochromatin likely plays a role in silencing of transposable elements and influences gene expression [155]. Compared to genetic diversity, little is known about epigenetic diversity in crops, which may prove an untapped reservoir of useful variation for crop improvement [156]. Some important initial results have been published recently. Daccord et al. [157] generated an improved genome assembly of domesticated apple and created a genome-wide map of DNA methylation. Their results hint at a potential role of epigenetic marks in the expression of agronomic traits in perennial fruit trees. Epigenetic regulation is of particular importance to study the relationship of subgenomes in polyploid crops such as wheat [158], cotton [159], and rapeseed [160].

Archaeogenetics—the extraction, sequencing, and analysis of ancient DNA fragments—has transformed our concepts of the history of human and animal species [161–163]. Until recently, plant genetics has not had access to this window into the past, mainly owing to a paucity of archaeobotanical remains containing sufficient amounts of well-preserved DNA [164, 165]. Recent studies in maize and barley retrieved DNA sequences from samples preserved under arid conditions and analyzed them together with sequences from extant individuals. da Fonseca et al. [166] thus reconstructed the past demography of maize in the southwest United States. Mascher et al. [167] reported a close genetic affinity of 6000-year- old barley grains from the Judean desert to present-day landraces from the Southern Levant. Swarts et al. [168] employed genomic prediction models trained on modern data to understand the temporal dynamics of adaption to temperate climates as maize cultivation spread northwards.

Genome editing with CRISPR-Cas9 technology [169] has enriched the plant geneticist’s toolkit [170]. The rapid induction of targeted mutations will be instrumental in validating putative domestication genes in the wild individuals and creating novel useful variation in the domesticate. For example, Soyk et al. [171] isolated two regulators of inflorescence architecture in tomato by means of map-based cloning and validation through classic mutants and CRISPR-Cas9 knock-out. Naturally occurring mutations in both genes had been independently selected either to increase fruit size or to facilitate mechanical harvesting. However, due to negative epistatic interaction between both genes, combinations of alleles that are beneficial on their own resulted in loss of fertility and excessive branching. Soyk et al. [171] then evaluated allelic series of both loci for epistatic interactions and found new beneficial allelic combinations that overcame negative epistasis. Braatz et al. [172] used CRISPR-Cas9 in tetraploid oilseed rape (*Brassica napus*) to knockout simultaneously both homeologs of *ALCATRAZ*, a known regulator of seed shattering in *Arabidopsis* [173]. Siliques of double mutants were partially resistant to shattering, an important trait to avoid seed loss during mechanical harvesting.

The domestication of new plant species has been proposed as an important future contribution to sustainable agriculture. For instance, the development of perennial grain crops has received considerable attention [174, 175]. Progress has been made in bringing intermediate wheatgrass (*Thinopyrum intermedium*; Fig. 1), a perennial relative of wheat, into cultivation with the establishment of dense linkage maps [131] and the implementation of genomic selection [176]. The domestication of bioenergy crops has been put forward to meet the growing demands for biofuel. Proposed targets include the aquatic fern *Azolla* [177], *Miscanthus* species [178], and the duckweeds [179].

As large population genomic datasets accrue in more species, analysis methods need to keep pace with the growing amount of input data. Efficient data structures have been devised to structure and handle large marker matrices [180, 181]. Imputation strategies to infer missing genotypes in low-coverage sequence data have been adopted in human genetics [182, 183]. Moreover, imputation methods, which take into account inbreeding or are geared towards experimental populations, have been developed specifically for plant genetics [184]. We expect genotype imputation to be widely used in plant genetic studies as comprehensive haplotype reference panels become available [185]. As an alternative or complement to the imputation of discrete allelic states, statistical uncertainties in genotype calling from shallow sequencing data can be recorded and considered during population genetic analyses [186, 187].

New algorithms have been developed to speed up traditional analysis methods such as principal component analysis [188, 189] and statistical inference of population structure [190]. These include flashpca [191], FastPCA [192], fastSTRUCTURE [193], ADMIXTURE [194], and sNMF [195]. Methods for understanding past demographic processes such as bottlenecks and migration events include genome-wide comparisons of allele frequencies from dense genomic marker datasets [196, 197], fitting coalescent models to whole-genome sequence data [100, 101], and computational environments for demographic simulations [198, 199].

In summary, progress in sequencing technology and analysis methods will make it possible to study the genetics and genomics of domestication in a wider range of crop species. In the coming years, chromosome-scale reference sequence assemblies and resequencing studies of large diversity panels will contribute to understanding the past and present diversity of domesticated plants and their wild relatives.

## Abbreviations

BAC: Bacterial artificial chromosome
GBS: Genotying-by-sequencing
Hi-C: Chromosome conformation capture (quantifies interactions between all possible pairs of fragments simultaneously)
RNA-seq: RNA sequencingSNP, Single-nucleotide polymorphism
